# Comprehensive Retinal Image Analysis for Aggressive Posterior Retinopathy of Prematurity

**DOI:** 10.1371/journal.pone.0163923

**Published:** 2016-10-06

**Authors:** Deepthi Rajashekar, Gowri Srinivasa, Anand Vinekar

**Affiliations:** 1 PES Center for Pattern Recognition, PESIT Bangalore South Campus, Bengaluru, Karnataka, India; 2 Department Of Computer Science and Engineering, PESIT Bangalore South Campus, Bengaluru, Karnataka, India; 3 Department of Pediatric Retina, Narayana Nethralaya Post Graduate Institute of Ophthalmology, Bengaluru, Karnataka, India; Justus Liebig Universitat Giessen, GERMANY

## Abstract

Computer aided analysis plays a nontrivial role in assisting the diagnosis of various eye pathologies. In this paper, we propose a framework to help diagnose the presence of Aggressive Posterior Retinopathy Of Prematurity (APROP), a pathology that is characterised by rapid onset and increased tortuosity of blood vessels close to the optic disc (OD). We quantify vessel characteristics that are of clinical relevance to APROP such as tortuosity and the extent of branching *i.e*., vessel segment count in the defined diagnostic region. We have adapted three vessel segmentation techniques: matched filter response, scale space theory and morphology with local entropy based thresholding. The proposed feature set equips us to build a linear discriminant classifier to discriminate APROP images from clinically healthy images. We have studied 36 images from 21 APROP subjects against a control group of 15 clinically healthy age matched infants. All subjects are age matched ranging from 33−40 weeks of post menstrual age. Experimental results show that we attain 100% recall and 95.45% precision, when the vessel network obtained from morphology is used for feature extraction.

## Introduction

Retinopathy of Prematurity (ROP) is a progressive disorder that affects premature infants of very low birth weight (<2000 grams), who have been exposed to high ambient oxygen concentrations such as in incubators [1]. The pathology is associated with five stages to describe the extent of damage or the narrow window of possible treatment. Of these, stages four and five typically translate to extensive damage that is almost impossible to treat/correct, whereas the first stage is too premature to initiate the treatment (as the disease may even regress on its own) [2, 3]. In this study, we investigate incidents of Aggressive Posterior ROP (APROP). APROP is characterised by rapid onset (hence, “aggressive”) and manifests in the posterior zone of the eye *i.e*., close to the optic disc (OD), also clinically called as ‘Zone 1’. The main characteristic of APROP is tortuous vessels in the posterior zone. Tortuosity is a measure of how twisted a vessel segment is. Higher tortuosity close to the OD is classified as severe APROP. We seek to diagnose the disease at its onset, in its mildest form.

A typical incidence of ROP progresses from stage 1 to stage 5 within a few weeks. In contrast, the rate at which APROP progresses to the fifth stage is significantly fast: APROP progresses to stage 5 within a few days. It is also called the “Rush disease” for the same reason [4]. Studies in South India show that 10.2% of the total infant population suffer from APROP while the occurrence of ROP is as high as 41.5% [5]. In particular, six districts of Rural Karnataka have reported APROP [4]. Furthermore, in North India, 40.70% of all APROP subjects were diagnosed with severity in the Zone 1 of the eye and 59.26% with severity in the clinical Zone 2.

Since APROP does not progress through stages in sequence, it is extremely important that the condition is noticed in good time and monitored regularly. To ensure early detection of the disease, following the guidelines from the Neonatal forum of India,

- all infants <28 weeks of gestation or
- all infants <1750 grams of birth weight,

are imaged as early as 2-3 weeks of age [6].

In India, each year one in twelve infants born is at the risk of vision impairment [7]. A majority of the incidences of such vision impairedness at birth has been observed in rural areas due to unscreened variants of ROP. Furthermore, the patient-doctor ratio is as high as 572,530.12 with only 400 retinal surgeons and 15 ROP specialists. It is needless to stress on how many subjects each doctor has to screen in order to successfully mitigate the risk of permanent blindness. To effectively diagnose, treat and monitor ROP, Karnataka Internet Assisted Diagnosis of Retinopathy of Prematurity (KIDROP) is India’s largest Tele-medicine initiative [8, 9]. Currently, KIDROP has established a process in which trained technicians image each subject (both eyes) in multiple focus angles *viz*., optic disc centered images, macula centered images and anterior eye image. Of these orientations, the optic disc and macula centered images are the easiest to obtain. The protocol prescribed to the technicians is to obtain and screen 7 images per eye for each subject [8]. If the technician decides that a subject requires attention, a medical practitioner then uses multiple images of all available orientations of the eye to diagnose the subject as APROP affected or clinically healthy. Since APROP prevails in the posterior region of the eye, an inexperienced technician might not notice the changes in vascular structures during examination, which might then lead to delayed diagnosis [4]. Computer aided analysis of eye structures ensures, that even the most circumferential symptoms are noticed at an early stage. A first round of aggressive screening on the considerable number of preterm babies is crucial, as we do not afford to ignore the early onset of APROP (mildest APROP incidence). The final decision on the diagnosis of APROP is done only by retinal specialists. On the other hand, given the incidence of APROP, doctors will have to screen many infants looking for symptoms of APROP. This diverts expert medical attention from cases that demand immediate care. Hence, developing a robust system to compute blood vessel tortuosity in each zone plays a vital role in the efficient and objective diagnosis of APROP, thereby avoiding a wrong diagnosis and permanent loss of sight in premature babies. As a first step towards such continuous monitoring and early detection, we have developed a system to identify fundus images in which subtle signs of APROP are evident.

Given a single image per eye (instead of 6-7 images as per the current screening protocol used by trained technicians), our system emulates the clinician’s decision for the subsequent screening by a qualified doctor. We envisage that these static tests, together with a study of the progression of the pathology would eventually lead to a robust system capable of performing an early detection.

### Background

The first step towards automated analysis involves delineating the vessel network in the eye. Vessel tracking, matched filter response, multiscale image analysis, morphological segmentation, region growing are some of the most cited approaches to vessel segmentation in literature [10]. However, they have not been investigated to diagnose APROP in particular. In this study, we focus our efforts on building a system to assist diagnosis of only APROP, without the co-occurrence of other retinal pathologies.

Medical practitioners agree that when APROP manifests with other retinal pathologies such as stage 2, stage 3 and smouldering ROP, specific symptoms of APROP are not evident.

The ARIA tool, developed by [11], proposes retinal image analysis with a system that automatically delineates vessel segments using wavelet transforms and analyzes vessel parameters such as vessel centerline refinement that is persistent with our problem. However, ARIA fails to recognize vessel loops in centerline detection and fails to distinguish vessels at proximal distances while detecting vessel edges around each vessel centerline. Vessel loops are significant contributors to vascular tortuosity and hence, it is important to segment them as a part of the vessel network. The merger of proximal vessels causes incorrect quantification of arborocity. A comprehensive system to automate retina vessel extraction and registration has been proposed based on vessel tracing techniques [12]. However, these often require user intervention to declare start and end points for the segmentation/ tracing.

RISA (Retinal Image multiScale Analysis) yields promising results in vessel segmentation, tortuosity and vessel width computation in retinopathy of prematurity [13]. It is certain that the nature of infant’s vessels are comparable in ROP and APROP. However, RISA requires users to manually select the vessel that needs to be segmented, traced and studied for tortuosity and dilation. A study that shares our ideology in computer aided diagnosis of retinopathy is one that relies on vessel tortuosity as a feature [14]. However, this system also requires user intervention to develop vessel masks for each fundus image which is also tedious and time consuming. Further, individual vessel tortuosities are highly appreciated in dynamic studies that record the rate at which a pathology aggravates.

Since our aim is to manage large scale screening of premature infants, we focus on minimising the manual intervention to compute average tortuosity *etc*., features from the input images. Moreover, our implementation of gaussian matched filter responses [15] enables us to get a vessel network which is symbolic of the extent to which vessels have developed in the fundus. The network thus obtained is not discontinuous and facilitates tortuosity computation. The scale space segmentation algorithm [16] ensures avoiding noise in the vessel network. Pixels representing the capillary and light artifacts are not misclassified as foreground pixels in this approach. Finally, morphological segmentation with local entropy thresholding allows us to obtain a network close to the ground truth. Proximal vessels and vessel cross overs are evident in this network. In particular, this algorithm ensures segmentation of the most prominent vessels in the fundus. Classification outcomes in the proposed morphological segmentation yield the desirable rates of a 100% recall and an acceptable false detection rate.

## Materials and Methods

### Data

The Narayana Nethralaya (NN) data set is obtained using a ROP lens in a RetCam shuttle, which ensures a 130° field of view specially designed for premature infants. All the subjects in this study were screened during 33-40 weeks (post menstrual age). The study has been approved by the Narayana Nethralaya Postgraduate Institute of Ophthalmology Research and Ethics committee and complies with the Helsinki Declaration of 1975, as revised in 2000. It is ensured that all subjects in the NN data set have been thoroughly de-identified. This study is retrospective and has not influenced the course of treatment of the patients in anyway.

**Fig 1:**
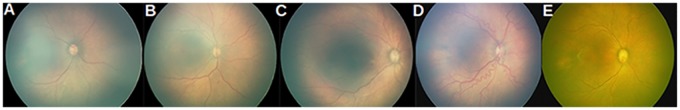
Representative NN data set. (a) Typical clinically healthy infant eye with immature vessels; (b) infant eye with apparent tortuous vessels in the nasal zone (left of the OD); (c) infant eye with developed vessels reaching the periphery of the eye; (d) severe APROP infant eye; (e) infant eye with APROP onset.

In practice, pre-term infants are screened at least once in the first 2-3 weeks of life, hence it is not difficult to obtain at least one set of healthy images of a premature subject. We have used a total of 36 fundus images for our study: 21 retinal images of subjects affected by APROP and 15 retinal images of clinically healthy subjects. Although the data set used in this study appear to be small, typical numbers used in ROP studies are 50 [17], 66 [13]*etc*. Standard datasets such as DRIVE [18] and HRF [19] 40 and 45 fundus images (adult eyes) respectively. The NN set is a representative one in that the clinically healthy set comprises of typical healthy infant eyes (see Fig 1(a)), infant eyes that have relatively matured vessels (see Fig 1(b)) and those that appear as mild APROP subjects as shown in Fig 1(c). The APROP set comprises images with severe and nascent stages of the disease as represented in Fig 1(d) and 1(e) respectively. It must be noted that the healthy sample in Fig 1(c) and the APROP sample in Fig 1(e) are much the same in terms of ‘extent of vessel growth’ and their corresponding tortuosity. Furthermore, despite belonging to the clinically healthy set, Fig 1(a) and 1(c) indicate a considerable variation in the rate of vessel maturity. That is to say, given that a premature infant is 33 weeks old (for example), one cannot predict and/or quantify the extent of retinal vascularisation accurately. It is therefore safe to conclude that the data set used in this study captures a wide spectrum of variations in each class *i.e*., clinically healthy and APROP.

### Proposed method

**Fig 2:**
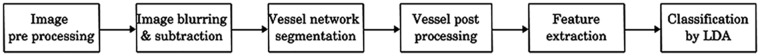
Proposed algorithm for vessel segmentation.

A block diagram of the proposed solution is shown in Fig 2.

In brief, the method comprises preprocessing, followed by segmentation, feature extraction and a rule-based classification. Since the goal of this work is to design a systematic approach to detect symptoms of APROP, we have instantiated the framework with three vessel network extraction techniques, to choose the method that best reflects features relevant to APROP. While the preprocessing steps remain similar, we have used different vessel postprocessing measures to ensure a fair comparison of the features across the three segmentation algorithms. The features used for designing the rule-based classifier for APROP are: the tortuosity index and absolute count of vessel segments.

### Image preprocessing

This step is intended to clear the images of artifacts, *etc*., that are likely to interfere with the feature computation step.

#### Saturation noise

**Fig 3:**
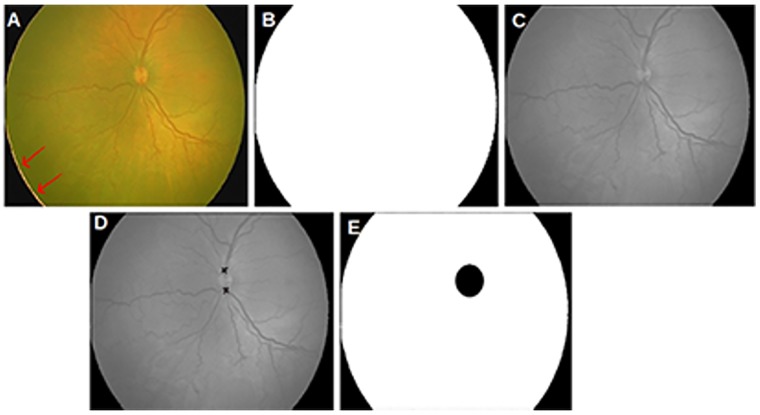
Stages of preprocessing. (a) Original image with arrows indicating saturation noise; (b) binary mask to remove saturation noise; (c) outer ring mask overlaid & saturation noise removed; (d) markers indicating major axis end points used to compute OD mask; (e) circular OD mask.

When imaging part of the retinal structure, illumination is well focused at the center of the region of interest (not at the image boundaries). This causes fundus images to have an outer ring of saturated pixels. In order to remove this saturation noise, a mask is computed for each eye, by thresholding the image to form a binary image. This is achieved by:

- equalising the histogram using the first and ninety ninth percentile of the intensity range, as the new minimum and maximum contrast stretch limits respectively.
- adjusting the global threshold value for the equalised image, according to the efficiency of thresholding.
- any holes resulting from thresholding are subsequently filled to obtain a good eye mask shown in Fig 3(b).

#### Optic disc mask

In most images, the OD has distinct boundaries. It is necessary to omit the OD boundaries in feature computation. The OD is not strictly circular. However, without loss of generality we use the major axis endpoints (see Fig 3(d)) to compute a circular mask of the OD. The mask thus obtained is shown in Fig 3(e) and is overlayed on the vessel network before proceeding toward feature extraction.

#### Channel selection

**Fig 4:**
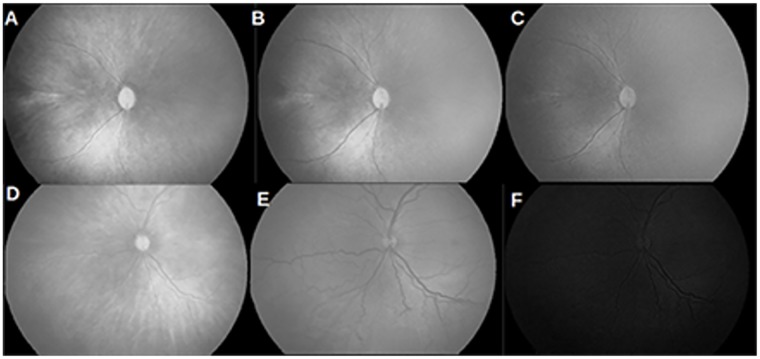
Relative uniformity in illumination across the red, green and blue channels respectively. (a-c) RGB color bands of a healthy fundus image, with blue being most uniform; (d-f) RGB color bands of an APROP fundus image, with the mean of blue channel <64.

As shown in Fig 4, the intensity variation in each channel of the RGB image is not similar in the clinically healthy and APROP image sets. While most healthy images have relatively uniform background illumination in the blue channel, the APROP posses relative uniformity in the green channel. In order to select the right color channel for further processing, we study the mean and variance values of each color band. First, we select those channels whose average intensity is greater that 25th percentile of the possible intensities *i.e*., a grayscale value 64. Of the selected channels, we choose the channel with least variance for further image analysis.

### Image blurring and subtraction

**Fig 5:**
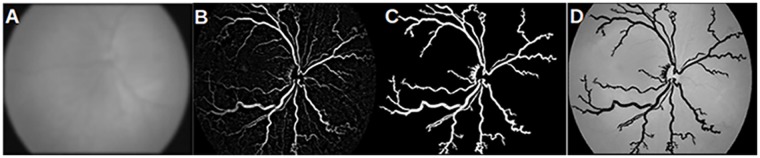
Stages in image blurring and subtraction. (a) vessel blurring; (b) subtraction from selected channel; (c) final segmented network; (d) segmented network overlaid in original image.

The selected channel of each image is convolved with a piece-wise linear unsharp mask of 9 pixels (empirically selected) across 360 degrees. Fig 5(a) shows the result of blurring all the vessels in the input image. When the blurred image is subtracted from the selected channel, we obtain an approximated grayscale vessel network as shown in Fig 5(b).

### Vessel network segmentation

Subsequently, the grayscale vessel network is thresholded based on local entropy for extracting a binary vessel network (see Fig 5(c)). The gray level co-occurrence matrix of the subtracted image is calculated. From the probability of co-occurrence of each gray value, the second order entropy is calculated for foreground and background pixels. Local entropy for the image is chosen to be the maximum of the sum of the foreground and background second order entropies [15]. Fig 5(d) shows the binary vessel network overlayed on original image. It is evident from Fig 5(d) that this method removes out of focus vessel segments.

### Vessel post processing

**Fig 6:**
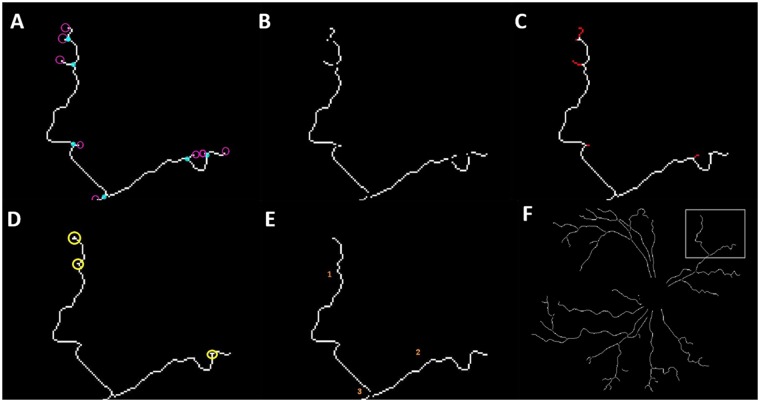
Vessel post processing. (a) branch points and end points indicated in blue and red respectively; (b) overlaying branch points results in pruned vessels; (c) terminal spurs indicated in red; (d) ‘L’ and ‘T’ shaped critical points leading to false end points; (e) subsequent thinning and branch points recomputed; (f) portion of the vessel skeleton in gray box zoomed in through (a-e).

Based on the strength of edges in various orientations, the binary vascular tree is of varying thickness. Thus, we use mathematical morphology to obtain a skeleton of the vascular tree of one pixel thickness. A portion of the postprocessed vessel network is shown in Fig 6(a).

A one-pixel wide blood vessel network enables us to locate branch points and compute end points as shown in Fig 6(b). When traversed through the vessel skeleton pixel-by-pixel, a branch point is one that has more than one route to take. Each branch point necessarily results in two or more vessel segments. An end point is a pixel of a vessel segment that has only one neighbour. For every segment in the vascular network two end points are computed. Vessel length is measured as the total number of pixels in each vessel segment. The thinned vessel network consists of terminal branches that are less than 10 pixels long. Overlaying branch points on the thinned vessels will cause vessels to split at the terminal branches (see Fig 6(c)), leading to incorrect computation of vessel length. This demands us to scan through end points and remove the terminal branches. We thin the network again to avoid detecting false end points as evident from Fig 6(d). Branch points and end points are recomputed, resulting in vessel segments devoid of spurs and terminal branches (see Fig 6(e) and 6(f)).

### Feature extraction

Since APROP is a pathology that manifests close to the OD, we define regions with reference to the OD, to compute features that are relevant to the pathology.

#### Defining diagnostic regions

**Fig 7:**
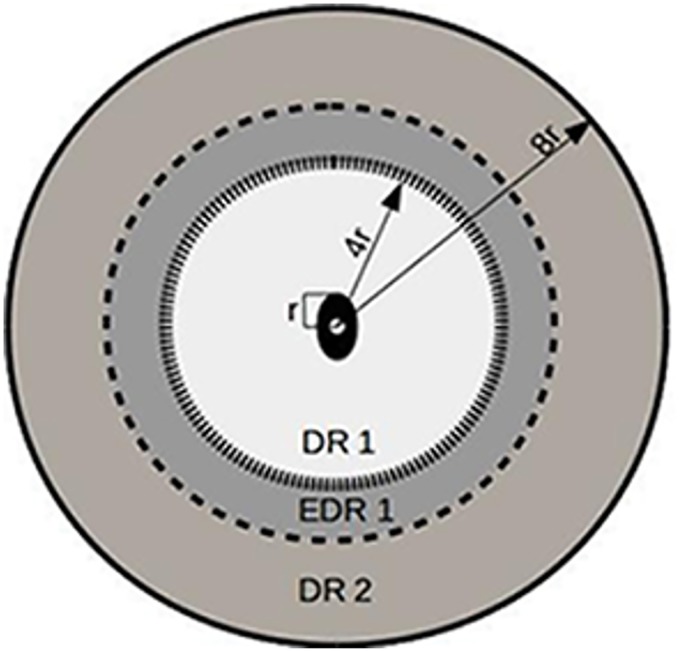
Pictorial representation of diagnostic regions (DR1, EDR1 & DR2).

The diagnostic regions (DR)’s are defined in terms of the OD radius (see Fig 7). DR1 is defined as the area 4*OD radius centered around the optic disc centroid. In order to lay more emphasis on vessel activity in the vicinity of the optic disc, we also define extended DR1 (EDR1) that stretches upto 6*OD radius. DR2 is defined as the area between [8*OD radius—4*OD radius] concentric to DR1 and centered at OD centroid. Anatomically it is observed, that the vessels from an arch approximately at 4*OD radius. As a consequence, tortuosity computation on these vessels yields a number that may actually be higher than taking the actual vessel length into consideration. In order to mitigate the spurious amplification of the average tortuosity index. We have defined an extended DR1 that is at distance of 6*OD radius. Thus any arching or branching at the boundary of DR1 does not contribute to the average tortuosity and hence labeling a healthy image as diseased. The same rationale is applied to the arches at the junction of EDR1 and DR2. Thus there is an annulus of overlap between DR1 and DR2 (EDR1).

This quantification helps us study the feature vectors from three perspectives: close to the optic disc (DR1); around DR1 (EDR1) and farther away from the optic disc (DR2). Since we study aggressive posterior retinopathy, our focus is on the vessel activity close to the OD which is predominantly in DR1 and EDR1.

It is to be noted that, all retinal images are not obtained at the same scale. However, these definitions of DR1, EDR1 and DR2 allow us to study comparable regions of the fundus image, around the OD across images with/without the pathology.

#### Tortuosity index

By definition, the higher the tortuosity index (*T*) of the vessel segment, the more twisted the segment is. Since no vessel is a perfect straight line. While a healthy vessel segment will have tortuosity index (*T*) greater than 1, it would be significantly higher for segments belonging to an APROP fundus.

$$T_{i}=\frac{1}{n}\sum_{j=1}^{n}\left({\frac{\lambda_{j}}{\left||\epsilon_{2j}-\epsilon_{1j}\right||}}_{2}\right),$$ (1)

where,

n: total number of vessels

*λ*: number of pixels in the *j*^*th*^ vessel

*ϵ*: endpoint of the *j*^*th*^ vessel

*T*_*i*_: tortuosity index of the *i*^*th*^ fundus image.

We define average tortuosity index of an image as, the mean of the 75th percentile of vessel tortuosities in the each eye. Naturally, it is expected that the *T* of a healthy image is lower than the *T* of an APROP image.

#### Segment count

Upon overlaying branch points, we count the resulting segments in the entire fundus. In comparison with healthy images, the APROP images exhibit excessive branching of vessels and hence a higher segment count. Our feature vector comprises of average tortuosity index of each eye and the corresponding segment count (*S*).

### Classification using LDA

A two class linear discriminant function is implemented to predict classification labels for all training images. Priors are set equally and we have used a symmetric cost function. This was preferred since the data set is small yet representative. The result is a decision boundary that separates all data points in the 2−*d* space such that the feature vectors classified as clinically healthy lie on one halfspace (one side of the decision plane) and those deemed APROP lie on the opposite side of the separating plane. We perform leave one out cross validation to obtain classification error rates for features computed from each of the afore mentioned segmentation techniques.

### Related methods for vessel segmentation

We have adapted methods from literature that correspond to studying vessels of various orientations and scales (resolutions).

#### Matched filter response

**Fig 8:**
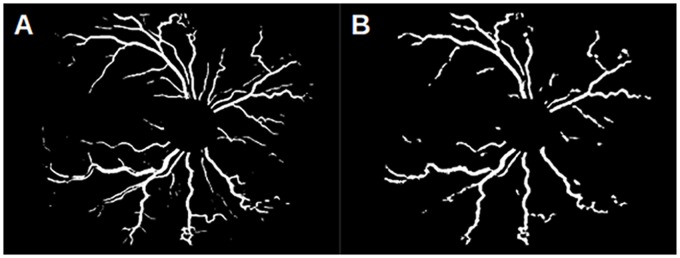
Vessel network extracted from. (a) matched filter response and (b) scale space segmentation.

We assume that vessels are aligned over the vertical axis and that the vessel gradients are symmetrical. Hence, it suffices to measure matched filter responses for each image pixel from 0 to *π*. A set of twelve kernels are defined to measure responses at fifteen degree intervals. The image is convolved with twelve kernels to obtain gradients along all fifteen orientations. Subsequently, of the twelve responses, only the maximum response for each pixel is retained. The raw response image from the matched filter is then thresholded by Otsu’s method (global threshold) to form a binary image of the vessel network (see Fig 8(a)).

#### Scale space theory

In this method, we pre-process the selected channel image across five scales *viz*., 3 × 3, 5 × 5, 7 × 7, 9 × 9 and 11 × 11. The gaussian blurred image in each scale is convolved with a laplacian edge detector. Subsequently, adaptive median filtering is done to ensure that capillary vessels do not get misclassified as vessel pixels, thereby also reducing noise. These images are thresholded and filtered to remove islands of white pixels. The final vessel network is the set of all pixels that are thresholded as foreground vessel pixels across three scales at minimum. The result of this approach is shown in Fig 8(b).

As reported by [20], due to a transparent retina in premature infants and low resolution of RetCam images, it is easy to confuse choroidal vessels from the vessel network that supplies blood to the retina. Scale space theory when applied to vessel segmentation ensures that, for a pixel to be classified as a ‘retinal vessel pixel’, it has to locally (a scale or window of interest) stand out as foreground pixel in at least three different resolutions. This avoids misclassification of choroidal vessels as retinal vessels.

### Adaptation

**Fig 9:**
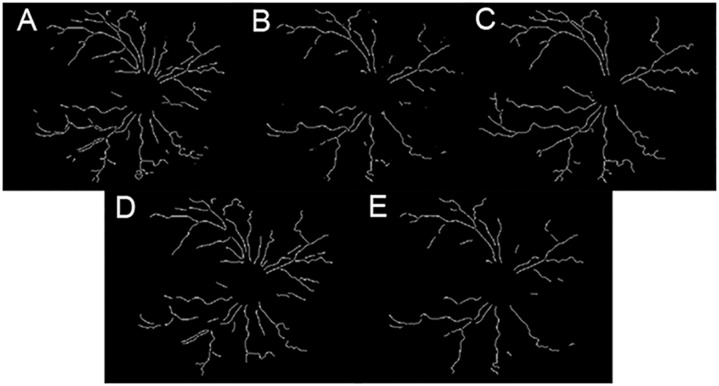
Vessel segments before area threshold. (a-c) prior to applying the area threshold (a) matched filter response; (b) scale space segmentation; (c) morphology with local entropy; (d-e) after the application of area threshold: (d) matched filter response; (e) scale space segmentation.

The foregoing approaches to vessel network extraction have been adapted to our data and problem statement *viz*., to segment ‘prominent’ vessels (ignoring vessel segments that are out of focus) from the diseased and healthy image sets. Fig 9(a) and 9(b) clearly indicates the presence of noise and insignificant vessel fragments. In order to ensure a fair comparison across the segmentation methods, we have appended another post processing step for the matched filter response and scale space algorithms.

The vessel segments obtained from the postprocessing undergo an area threshold. This ensures that the algorithm does not include short fragments in feature vector computations. It is apparent that the segments obtained from matched filter response and scale space segmentation in Fig 9(d) and 9(e) are now comparable to the set obtained from morphology (see Fig 9(c)). In the next section, we discuss the efficiency of features computed from all three methods of segmentation *i.e*., segment sets from Fig 9(d), 9(e) and 9(c).

## Results and discussion

### Comparison of features across segmentation methods

Analysis of variance (ANOVA) plots are used to indicate the difference in the mean *T* and *S* for each class being studied. We can also infer the variation of each feature about its mean value.

#### Tortuosity index

**Fig 10:**
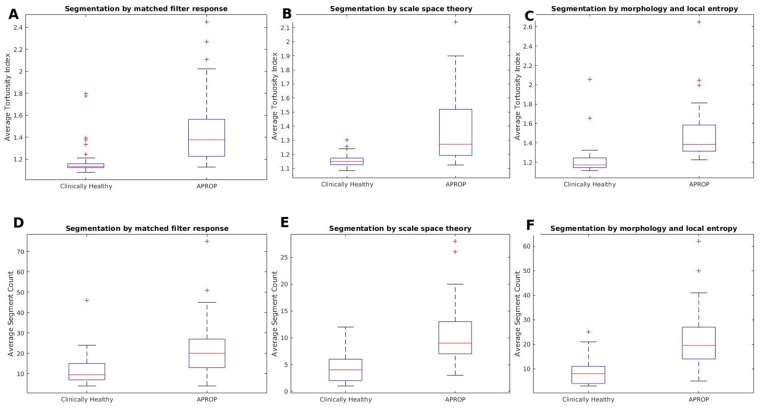
Anova plots. (a-c) Anova plots comparing *T* feature in (a) matched filter response; (b) scale space segmentation; (c) proposed method and (d-e) Anova plots comparing *S* feature in (d) matched filter response; (e) scale space segmentation; (f) proposed method.

It is evident from Fig 10(a) and 10(b) that the matched filter responses and scale space method have considerable overlap in the variance of *T* values of APROP class and those of the healthy class. However, the proposed method results in a better separation with *T* as a standalone feature (see Fig 10(c)).

#### Segment count

From Fig 10(d)–10(f), we can conclude that the proposed segmentation method results in the best separation by number of segments in the eye. APROP images result in a mean segment count that is greater than the maximum segment count in the healthy set. Furthermore, misclassified capillary pixels in the matched filter response leads to significant overlap in segment count variance about the mean *S* (see Fig 10(d)).

### Classification results

We obtain two linear discriminant functions applied independently to the following regions of interest:

- Diagnostic region 1 of optic disc centered images (OD DR1) and
- Extended diagnostic region 1 of optic disc centered images (OD EDR1)

**Fig 11:**
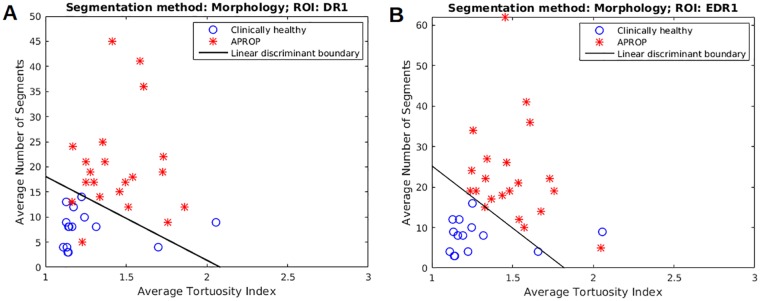
Result of LDA classification on OD centered images. (a) OD DR1; (b) OD EDR1.

The separating boundaries thus obtained, have been overlayed to present the predicted labels for OD centered images (Fig 11(a) and 11(b)). Our system deems a subject to be clinically healthy only if neither region of interest (*i.e*., DR1 and EDR1) of the eye gets classified as APROP. This measure ensures that no APROP subject gets erroneously misclassified as healthy and hence timely medical intervention. Although we allow a false positive (FP), our method results in zero false negatives (FN).

The table for classification error rates using all three approaches to vessel segmentation (both orientations) is given in Table 1. Features computed from vessel network obtained by morphological segmentation, yield 100% recall with 95.45% precision.

**Table 1:** Classification results for each orientation and corresponding region of interest.

Orientation	Matched filter response		Scale space theory		Morphology	
	Recall (%)	Precision (%)	Recall (%)	Precision (%)	Recall (%)	Precision (%)
DR1	85.71	90	85.71	100	90.47	95
EDR1	85.71	90	85.71	100	95.23	95.23
FINAL	85.71	90	85.71	100	**100**	**95.45**

### Misclassifications in APROP class

**Fig 12:**
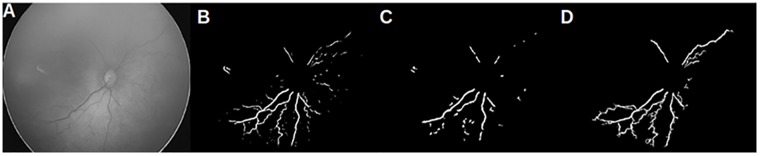
Vessel networks of a FN. (a) Original APROP image; (b) vessel network from matched filter response; (c) vessel network from scale space theory; (d) vessel network from proposed morphology.

One of the APROP subject is misclassified as healthy by vessel networks obtained from matched filter response and scale space theory. Fig 12(b) shows the noisy network obtained from matched filter response and the network from scale space theory is discontinuous (see Fig 12(c)). Segmentation by morphology results in a rather continuous and clean vessel network. In comparison with matched filters and scale space theory, the amount of vessel network segmented by morphology is also significantly high. This allows the proposed model to rightly classify the APROP subject.

### Misclassifications in clinically healthy class

**Fig 13:**
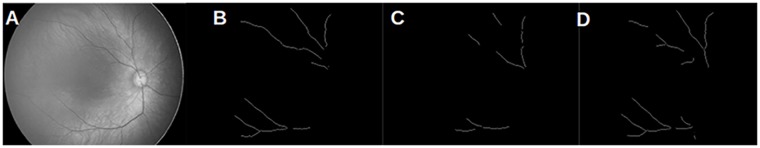
Vessel networks of a FP. (a) Original healthy image; (b) vessel network from matched filter response; (c) vessel network from scale space theory and (d) vessel network from proposed morphology.

**Fig 14:**
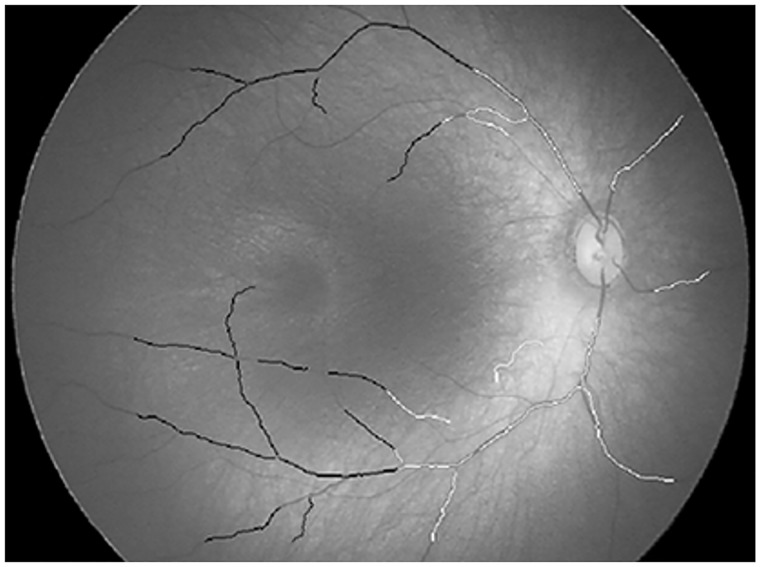
Healthy image overlaid with vessel segments in DR1 (white) and DR2 (black).

The suggested method to vessel extraction results in one FP. The infant eye in Fig 13(a) is incorrectly classified as APROP owing to the increased segment count. In a matured eye, the vessels branch and grow to the periphery of the eye. Since each branch is long enough not to be deemed as a terminal spur, this subject is assigned a higher *S*. Another example of matured retinal vasculature is shown in Fig 14. The vessels belonging to DR2 (indicated in black) are seemingly tortuous. The extent of vessel growth is subjective to the infant’s growth rate. Therefore, we cannot establish a standard threshold for *S* and *T* per class. Since vessel activity in DR1 (and EDR1) is normal, we infer that the subjects are not suffering aggressive ‘posterior’ retinopathy of prematurity. However, they should be screened for the onset of other retinal pathologies that sideline APROP. Hence, the system is designed to detect even the slightest manifestation of symptoms of APPROP manifestation of APROP.

### Vessel activity in DR2

**Fig 15:**
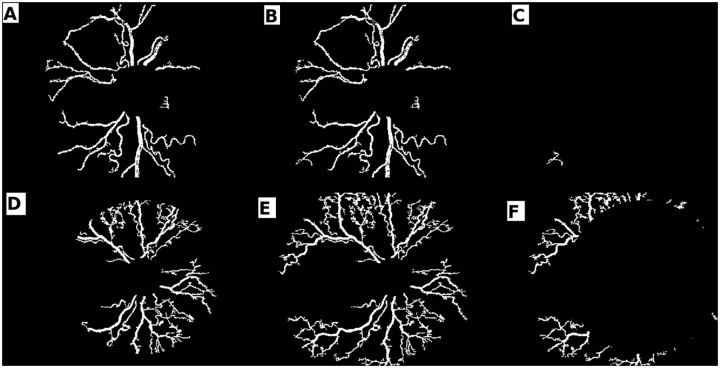
Varying levels of arborocity in APROP. (a-b,d-e) vessel segments in DR1, EDR1 and DR2 respectively; (c) APROP sample without vessel segments in DR2; (f) APROP sample with too many vessel segments in DR2.

**Fig 16:**
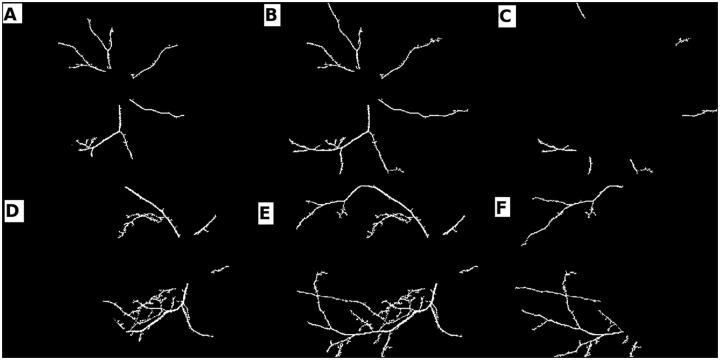
Varying levels of arborocity in healthy. (a-b,d-e) vessel segments in DR1, EDR1 and DR2 respectively; (c) Immature clinically healthy sample; (f) Mature clinically healthy sample.

Arborocity of vessels in the vascular retina spell symptoms of retinal neovascularisation. When observed in the peripheral retina (our DR2), this feature represents the manifestation of plus disease, which is a predecessor to the onset of APROP. We thus proceeded to study vessel behaviour in DR2 and the following observations were made:

- An APROP subject could not have vessel growth in DR2, indicative of capillary non-perfusion [21] (see Fig 15(c)).
- An APROP subject could have too many short and tortuous vessel segments in DR2, indicative of neovascularisation (see Fig 15(f)).
- A healthy subject with an immature retina could not have vessels grown to the extent of DR2 (see Fig 16(c)).
- A healthy subject with a mature vascularised DR2 (see Fig 16(f)).

However, arriving at a viable estimate for vessel branching, in healthy premature infants would be impossible given the limited availability of data pertaining to the clinically healthy set. It might be worthwhile to screen images classified as clinically healthy (with a vascularised peripheral retina) for other variants of ROP. However, this remains outside the scope of our preliminary study for ‘posterior ROP’ which is apparent in regions close to the OD (DR1 and EDR1).

## Conclusion

**Fig 17:**
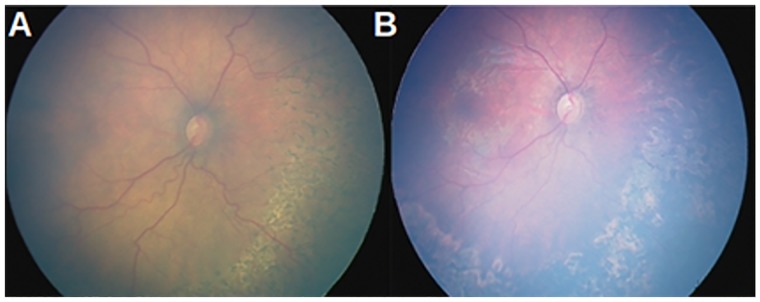
Severe APROP subject treated post diagnosis. (a) vessels after first sitting of laser burns; (b) vessels with reduced tortuosity after second week of laser therapy.

The NN data set used in this study is fairly representative, *i.e*., from a mild manifestation of APROP through symptoms that could easily be overlooked by a novice and mistaken as healthy (see Fig 1(e)) to a severe manifestation of APROP as shown in Fig 1(d). Such a severe case is also treated using laser therapy once diagnosed with APROP (see Fig 17 for instance). The tortuosity was found to have decreased through stages of the treatment. Thus, this static study enabled us to classify an eye as healthy or diseased, requiring further stages of clinical assessment and treatment. In the future, we intend to add other clinically relevant features that will allow us to conduct a dynamic study *i.e*., to predict the onset of the pathology.
